# Exploring the Impact of Online Medical Team Engagement on Patient Satisfaction: A Semantic Features Perspective

**DOI:** 10.3390/healthcare12111113

**Published:** 2024-05-29

**Authors:** Siqi Wang, Xiaofei Zhang

**Affiliations:** Business School, Nankai University, Tianjin 300071, China; siqiwang01@163.com

**Keywords:** online health communities, online medical team, patient satisfaction, team engagement, semantic feature

## Abstract

Online medical teams (OMTs), a new mode of online healthcare service, have emerged in online health communities (OHCs) in China. This study attempts to explore the underlying mechanism of how OMTs’ engagement influences patient satisfaction through the lens of semantic features. This study also scrutinizes the moderating effect of multiple specializations on the link between OMTs’ engagement and semantic features. We utilized a linear model that had fixed effects controlled at the team level for analysis. A bootstrapping approach using 5000 samples was employed to test the mediation effects. The findings reveal that OMTs’ engagement significantly improves language concreteness in online team consultations, which subsequently enhances patient satisfaction. OMT engagement has a negative impact on emotional intensity, ultimately decreasing patient satisfaction. Multiple specializations strengthen the impact of OMT engagement on both language concreteness and emotional intensity. This study contributes to the literature on OMTs and patient satisfaction, providing insights into patients’ perceptions of OMTs’ engagement during online team consultation. This study also generates several implications for the practice of OHCs and OMTs.

## 1. Introduction

Online health communities (OHCs) have become a popular platform through which patients can access health information, seek professional advice, and receive social support [1,2,3,4]. In recent years, China’s OHCs have witnessed the emergence of online medical teams (OMTs), a novel mode of online health service [5]. OMTs enable doctors to collaborate virtually, offering online team consultations (OTCs) to patients [6]. By harnessing the collective expertise of multiple doctors, OMTs aim to tackle complex health issues [7]. However, the variability in OMT engagement poses uncertainty regarding the extent to which OTCs meet patient needs. Team engagement, reflecting the collective involvement of team members in collaborative tasks [8], varies across OTCs and can significantly impact their quality [9]. While some OTCs involve a single doctor, others engage multiple professionals, leading to differences in service performance. However, how OMTs’ engagement influences patient outcomes remains unclear. To address this gap, this study explores the impact of OMT engagement on patient satisfaction, a crucial indicator of online consultation quality [10].

In addition to the importance of team engagement for patient satisfaction, how team engagement affects satisfaction is also notable. This study examines how the OMT’s semantic features during OTCs mediate the relationship between OMT engagement and patient satisfaction. Semantic features are the specific textual meanings, such as actions, objects, and emotions conveyed [11,12]. In this study, we specifically focused on two crucial semantic features: language concreteness and emotional intensity, which represent the cognitive and emotional dimensions of communication, respectively [13]. Language concreteness refers to the phenomenon that the same content can be expressed using varying levels of concreteness [14]. Language concreteness, determined by the specificity of message content [14], varies from detailed to general descriptions. Concrete descriptions reveal more detail and abstract descriptions are more general and require more inference [13]. For example, in a medical setting, the terms “sphygmomanometer” and “medical device” can be used to describe the same object, but the former is more concrete than the latter and makes it easier for people to understand. Emotional intensity refers to the proportion of expressed emotions in communication [14]. There may be different levels of emotional content in doctor-patient communication [10]. In OTCs, the difference in OMT engagement might significantly influence the semantic content that the team expressed. We posit that a greater extent of OMT engagement can increase language concreteness and emotional intensity, which, in turn, improves patient satisfaction. Hence, the first research question addressed in this study is:

*RQ1: Does OMT engagement improve language concreteness and emotional intensity during OTCs, which subsequently enhances patient satisfaction?*

Further, the impact of OMT engagement on language concreteness and emotional intensity may be influenced by the characteristics of doctors who are involved in the OTC. Multiple specializations are a likely important factor. Multiple specializations reflect collaborative efforts among doctors who have diverse knowledge and skills, effectively addressing patients’ health concerns. During team consultations, specialists from various departments offer unique perspectives, which can facilitate the overall team performance [15]. Accordingly, this may enhance the effectiveness of OMT engagement, reflected in the improvement in language concreteness and emotional intensity. To explore the possible positive effect of multiple specializations on the relationship between OMT engagement and team semantic features, our second research question is:

*RQ2: Is the impact of OMT engagement on language concreteness and emotional intensity contingent on multiple specializations?*

To address the aforementioned research questions, we conducted an empirical investigation by collecting data from a leading OHC in China. Our analysis is based on the data set that includes 92,946 consultation records. The findings reveal that OMTs’ engagement significantly improves language concreteness in OTCs, which subsequently enhances patient satisfaction. OMT engagement has a negative impact on emotional intensity, ultimately decreasing patient satisfaction. Multiple specializations strengthen the impact of OMTs’ engagement on both language concreteness and emotional intensity. Our study is one of the few to investigate the impact of OMTs’ engagement on patient satisfaction in the context of OTCs, shedding light on the mediating role of OMTs’ semantic features. Our findings contribute to the understanding of factors that drive patient satisfaction during OTCs, expanding the literature on OMTs and patient satisfaction. Further, this study holds important implications for OHC and OMT practitioners.

## 2. Literature Review

### 2.1. Online Medical Teams and Team Engagement

In recent years, the development of OMTs has introduced a novel approach to online healthcare services. Studies have revealed that joining OMT can benefit the performance of member doctors [16,17]. Moreover, scholarly attention has also been paid to the factors that contribute to high-performance OMT, such as knowledge collaboration [6]. Notably, team diversity has garnered considerable interest. Various forms of diversity within teams have been linked positively to OMT performance, encompassing reputation diversity, experience diversity [5], gender and hospital diversity [18], and professional knowledge diversity [19]. Despite the advantages offered by OMTs in facilitating collaboration among specialists [7], concerns persist regarding the level of engagement during consultations. For example, in some consultations, there is only one doctor who provides answers to the patient while other doctors remain silent. Such limited engagement may lead to inadequate information exchange and emotional support, ultimately compromising the efficacy of patient care and potentially impacting health outcomes negatively. Enhanced engagement among member doctors is therefore pivotal for augmenting the quality of team-based services, as it facilitates the pooling of diverse knowledge and skills into collective medical efforts. Recognizing the significance of OMT engagement, it becomes imperative to investigate its impact during OTCs.

Given the growing emphasis on team outcomes, effective teamwork necessitates broader member engagement [9]. Team engagement emerges as a critical factor influencing overall team performance [20,21]. The engagement of a medical team has been shown to help reduce the stress of the work and improve professional performance and the quality of provided care [22]. Conversely, inadequate team engagement may result in adverse consequences, including diminished advice accuracy and comprehensiveness [23] and an increased likelihood of incorrect medical decisions [24]. Accordingly, understanding the dynamic of OMTs’ engagement during OTCs is crucial for ensuring the quality of collective medical service, as well as enhancing the overall patient experience. In this light, this study aims to scrutinize the impact of OMTs’ engagement in online consultations.

### 2.2. Patient Satisfaction

Given the increasing importance of patient-centered care, enhancing patient satisfaction has become a pivotal concern in delivering healthcare services [25]. High levels of patient satisfaction have been found to be associated with improved compliance [10], promoted emotional and physical well-being [26,27], and decreased malpractice claims [28]. Satisfaction is a criterion for the extent to which healthcare providers meet patients’ needs, expectations, and preferences [29,30]. Additionally, satisfaction is a valuable indicator of the quality of interpersonal relationships between patients and their doctors [31]. The factors that influence patient satisfaction, such as service price [32], response speed and interaction frequency [33], and social support [34], have been studied extensively. However, how the semantic features of OMTs can affect patient satisfaction remains unexplored, especially in terms of the impact of OMTs’ engagement. To address this gap, this study scrutinizes the effect of OMTs’ engagement on patient satisfaction through the mediating role of OMTs’ semantic features.

## 3. Development of Hypotheses

**Figure 1:**
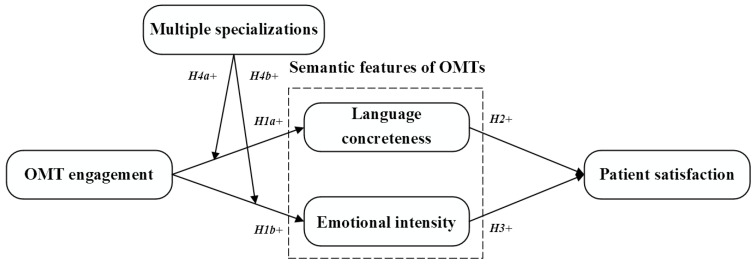
Research model.

This study explores the impact of OMTs’ engagement on patient satisfaction. Using the mediating role of language concreteness and emotional intensity, we constructed a research model (Figure 1). In the model, language concreteness and emotional intensity embody the semantic features of OMTs during OTCs. The moderating role of multiple specializations is also scrutinized in the model.

The success of a team depends on the interactions among its members in pursuit of shared goals [35]. Enhancing medical engagement is perceived as a key contributor to delivering high-quality and secure health services [36]. Prior studies have affirmed the favorable effects of team engagement on various aspects of team performance [20,21]. Increased engagement within a team signifies heightened dedication and commitment, thereby improving team effectiveness [37]. In addition, team engagement plays a pivotal role in cultivating a positive team environment, facilitating the generation and assimilation of new ideas that boost overall performance [37,38]. When more doctors are engaged in the consultation, they can more meticulously analyze the patient’s health problems and provide a more nuanced and perspicuous interpretation. Therefore, the overall language concreteness may be improved as a result. In addition, a higher level of team engagement allows more doctors to provide emotional support to patients, which also increases the emotional intensity of the consultation. In light of this, we posit the following hypotheses:

**Hypothesis 1a.**  *OMTs’ engagement has a positive impact on language concreteness.*

**Hypothesis 1b.**  *OMTs’ engagement has a positive impact on emotional intensity.*

Concrete language is considered more vivid and realistic than abstract language [39], as well as more likely to build trust in communication [40]. Given that professional and official medical information is generally abstract, patients may not fully understand the doctor’s response during the consultation [41]. More concrete language can improve the understandability of information, thus making it easier for patients to understand the health information provided by the OMT [42]. Therefore, in our research context, increased language concreteness in OTCs helps patients in fully understanding their disease and the doctor’s recommendations for self-management, in turn making patients more likely to be satisfied because their questions have been answered clearly and well.

The unhealthy state of patients can be accompanied by anxiety, depression, worry, and other negative emotions that can have a negative effect on their self-management and health conditions [10]. A greater extent of emotional intensity can provide more emotional support to the patients, demonstrating the doctors’ empathy, and is more likely to increase the reported satisfaction [43,44]. Thus, we believe that a higher level of emotional intensity embedded in the communication content may provide patients with a more positive emotional experience, thus alleviating their negative emotions and, in turn, leading to higher satisfaction.

**Hypothesis 2.**  *Language concreteness has a positive effect on patient satisfaction.*

**Hypothesis 3.**  *Emotional intensity has a positive effect on patient satisfaction.*

In this study, we define multiple specializations as the involvement of doctors from different departments in OTCs. In healthcare, where patients often present with complex conditions, undergoing a team consultation with a range of skills and knowledge ensures a more comprehensive and accurate approach to diagnosis and treatment [23]. In medical teams, the involvement of staff members from different departments proves beneficial in averting medical errors and enhancing performance [19]. Collaborating across diverse backgrounds taps into a broader spectrum of skills compared to homogeneous collaboration, thereby refining final decisions [45]. A multidisciplinary approach provides diverse professional knowledge, fostering creativity and innovation by encouraging the exchange of ideas from different fields. This approach can lead to novel solutions to medical problems, improve treatment, and enhance problem-solving [45,46,47]. Moreover, specialization can facilitate knowledge integration and enrich task-related knowledge to deal with medical challenges [48,49]. Accordingly, the language concreteness and emotional intensity during OTCs may be amplified when doctors from diverse departments are involved. Hence, we posit the following:

**Hypothesis 4a.**  *Multiple specializations have a positive impact on the relationship between OMT engagement and language concreteness.*

**Hypothesis 4b.**  *Multiple specializations have a positive impact on the relationship between OMT engagement and emotional intensity.*

## 4. Methodology

### 4.1. Research Context and Data Collection

Data for this study were sourced from Haodf (haodf.com), a prominent OHC in China. Haodf was launched in 2006 and has attracted over 240,000 physicians, serving more than 79 million patients by July 2023 (https://www.haodf.com/info/aboutus.php, accessed on 18 April 2024). The OMT service on Haodf commenced in 2017, allowing doctors to initiate their own OMT or join existing OMTs formed by their peers. Patients can access and buy team consultation services through a doctor’s homepage on which a link to the OMTs that the doctor has joined is provided. The collected data encompass dialogue records of OTCs and information at the team level. After cleaning up the data that had missing values, our data set includes 2122 OMTs, comprising 92,946 consultations. The time scope of our investigation spans from 2017 to 2023.

### 4.2. Measures

The dependent variable in this study is patient satisfaction (Patient satisfaction). Given the OHC does not provide an official module to enable patients to evaluate the OTC services, we choose to measure satisfaction through text analysis. Specifically, we adopted a customized satisfaction dictionary [10]. This dictionary aims to measure patient satisfaction in online consultations in the Chinese context. It includes the words that patients use to express their recognition to their doctors (e.g., “professional”, “reliable”, “enthusiasm”, and “carefulness”). We calculate the total number of satisfaction words to measure patient satisfaction. The independent variable in this study is OMTs’ engagement (OMT engagement), which reflects doctors’ involvement during consultations. We measure it by dividing the number of doctors who engaged in an OTC by the team size (i.e., the number of doctors in an OMT). This metric effectively captures the extent to which an OMT is devoted to the consultation. The mediating variables are language concreteness and emotional intensity. To calculate the average language concreteness in each OTC, we divided the sum of concreteness ratings for words by the total word count of doctors’ replies. The calculation of the total concreteness rating of OMTs’ expression in each OTC is based on a dictionary of approximately 40,000 commonly used English words and expressions obtained via internet crowdsourcing [50]. This dictionary rates these words and expressions on a five-point scale from abstract to concrete. We chose this dictionary for its specific focus on language concreteness and its demonstrated reliability and validity. Additionally, it encompasses both medical and everyday vocabulary and has been utilized to measure language concreteness in medical contexts [14,51], making it appropriate for measuring concreteness in OTCs. Given that our data are obtained from a leading OHC in China, we utilize the Chinese version of this dictionary [10]. The emotional intensity of each OTC is calculated by dividing the total emotional words by the OMT’s total word count. We use Linguistic Inquiry and Word Count (LIWC) 2022 to analyze the emotional content in OTCs. LIWC, which has been widely used to identify the emotional content in the online health context, provides a dictionary that detects positive and negative emotional words [52]. In accordance with prior research, we compute the total emotional words by summing positive and negative emotional words [14]. Multiple specializations reflect the diverse knowledge possessed by OMT members. Thus, multiple specializations are marked as 1 if the doctors who responded to the patients are from different departments and 0 if the doctors are from the same department. The departments contained in the data set include medicine, surgery, gynecology and obstetrics, pediatrics, osteological surgery, ophthalmology, oral science, otolaryngology, oncology, dermatology, andrology, burn, traditional Chinese medicine, infectious disease, nutritional sciences, and general practice. We also incorporate patients’ sex (Sex) and age (Age) as control variables into the model. Table 1 presents the descriptive statistics of the main variables of this study.

### 4.3. Analysis and Results

Our analysis is conducted in two stages. In the first stage, we examine the effect of OMTs’ engagement on language concreteness and emotional intensity and the moderating effect of multiple specializations. The models are hierarchically constructed and tested. Models 1 and 4 include only control variables; Models 2 and 5 incorporate control variables, independent variables, and moderating variables; and Models 3 and 6 explore the moderating effects by integrating the interaction terms. In the second stage, we test the effects of language concreteness and emotional intensity on patient satisfaction, starting with a baseline model that includes all the control variables. Then, we examine the impact of language concreteness and emotional intensity on patient satisfaction in model 2. To avoid the bias that may arise from differences among OMTs, we use linear regression that includes fixed effects controlled at the team level for analysis. STATA 17 is used to run these models.

The results of the first stage are shown in Table 2. From Model 2, we can see that OMT engagement is positively associated with language concreteness (β = 0.025, *p* < 0.1), which supports H1a. However, we find that OMT engagement has a negative impact on emotional intensity (β = −0.005, *p* < 0.01), contradicting our H1b that OMT engagement positively impacts emotional intensity. For the moderating effect, we find that multiple specializations strengthened the impact of OMT engagement on language concreteness (β = 1.412, *p* < 0.01) and emotional intensity (β = 0.915, *p* < 0.01); thus, H4a and H4b are supported. Table 3 presents the results of the second stage. We can observe that language concreteness (β = 0.097, *p* < 0.01) and emotional intensity (β = 2.624, *p* < 0.01) positively and significantly influence patient satisfaction; thus, H2 and H3 are supported.

Further, we examine the mediating effects of language concreteness and emotional intensity through a bootstrapping method with 5000 replications. The results show that the 95% bias-corrected confidence intervals (CI) of all the indirect pathways exclude zero (Table 4), indicating that the mediation effects are significant. This shows that OMT engagement improves patient satisfaction by facilitating language concreteness during OTCs while impairing patient satisfaction by decreasing emotional intensity.

### 4.4. Robustness Checks

We also conduct several additional tests to ensure the robustness of our findings. First, we use the ordinary least square model (OLS) to reexamine our model. The results are shown in Table 5 and Table 6, which demonstrate consistency with previous models. Second, we add new control variables to the model, including the OMT’s service price (Price), team size (Team size), whether the team leader has engaged in the consultation (Leader response), and the number of years since an OMT was formed (Tenure). The results, as depicted in Table 7 and Table 8, align consistently with our prior models. Hence, we are more confident about our findings.

## 5. Discussion

### 5.1. Main Findings

Our findings reveal that OMTs’ engagement significantly improves the team’s language concreteness in OTCs, which subsequently enhances patient satisfaction. With greater team engagement in OTCs, there emerges a heightened ability to meticulously analyze patients’ health concerns and provide more nuanced explanations. Consequently, overall language concreteness and precision see improvement. Subsequently, this refined language clarity empowers patients with a deeper and clearer comprehension of the medical information relayed during OTCs, culminating in heightened levels of patient satisfaction [10].

Contrary to our expectations, OMT engagement appears to have a detrimental effect on emotional intensity, consequently diminishing patient satisfaction. This finding suggests a distinctive dynamic in OMT services, emphasizing information-based interactions. In OTCs, doctors consistently deepen and refine discussions about health issues, enhancing the specificity of language and thereby generating more accurate treatment recommendations [23]. However, this emphasis on medical detail may inadvertently sideline emotional support in OTCs. As a result, reduced emotional intensity during these consultations may compromise patient satisfaction, as patients feel their expressions of concern receive inadequate positive feedback, such as reassurance [10,42].

Further, we find that multiple specialization strengthens the impact of OMT engagement on both language concreteness and emotional intensity. Embracing diverse specializations empowers doctors to enrich their medical advice with unique insights, fostering nuanced and concrete communication that enhances patient comprehension. Moreover, a multifaceted professional background equips doctors with varied insights into patients’ experiences of pain, enabling them to offer empathetic support from diverse angles [53].

### 5.2. Theoretical Implications

This study contributes valuable insights to the existing literature. First, this study is the first to examine the impact of OMT engagement on patient satisfaction, enriching the literature on OMTs and patient satisfaction. Although prior research has extensively studied the factors that influence the performance of OMTs [5,18,19], no study has examined how patients evaluate the OMT service. This study emphasizes the importance of team engagement in improving patient satisfaction during OTCs. Enhanced OMT engagement not only fosters broader medical collaboration but also ensures the provision of more comprehensive treatment recommendations [23]. Highlighting the significance of OMT engagement advances the development of patient-centered healthcare, thereby enhancing scholarly insights into OMT services, a novel mode of online health services.

Second, this study pioneers the exploration of how OMT engagement influences patient satisfaction through the lens of semantic features. The current understanding of how team engagement affects semantic features in OTCs remains insufficient. By delving into language concreteness and emotional intensity, this study unveils two distinct pathways explaining how OMTs’ engagement impacts patient satisfaction. Our findings indicate a contrasting dynamic between the cognitive and emotional aspects of OMT-patient communication. Increased OMT engagement fosters a cognitive-focused approach, where doctors prioritize addressing health issues, as evidenced by enhanced language concreteness during OTCs. However, this emphasis may concurrently diminish emotional intensity, leading to reduced patient satisfaction. By shedding light on cognitive and emotional mediators, our findings contribute to a deeper understanding of how OMT engagement influences patient satisfaction through the semantic features in OTCs.

Third, our findings reveal the importance of multidisciplinary member contributions during OTCs. Although previous research has indicated that OMT diversity can benefit team performance [5], how the multidisciplinary collaboration within OMT generates positive outcomes remains unknown. The participation of multidisciplinary doctors enhances both the language concreteness and emotional intensity during OTCs, thereby fostering patient satisfaction. Moreover, this collaborative approach fosters a more holistic and precise diagnosis and treatment strategy by promoting the synthesis of diverse expertise [23,48,49]. This integration paves the way for innovative solutions to medical challenges and amplifies problem-solving capabilities [45,46,47]. Acknowledging the benefits of multidisciplinary engagement can elevate the effectiveness of OTCs and the value of OMT services, particularly when addressing intricate health issues [54].

### 5.3. Practical Implications

Our findings also hold valuable implications for practice. First, our findings highlight the importance of the engagement of OMT members in enhancing patient satisfaction during OTCs. Accordingly, OHC managers can take actions to facilitate OMTs’ working engagement, for example, by implementing incentive policies. Furthermore, it is crucial for platforms to value the semantic content conveyed during OTCs, as heightened levels of language concreteness and emotional intensity correlate with increased patient satisfaction. It is necessary for OHCs to encourage doctors to employ concrete language to aid patients in comprehending their medical status and recommendations effectively. Additionally, recognizing that patients often perceive medical teams as vital sources of emotional support, OMTs should actively offer emotional support during consultations, as this significantly contributes to overall patient satisfaction. Neglecting this aspect of emotional interaction risks leaving patients dissatisfied.

Second, OHC managers should recognize that multiple specializations can amplify the impact of OMT engagement on language concreteness and emotional intensity during OTCs. Hence, facilitating the formation of multidisciplinary teams may generate better influence on the platform compared to teams that are homogeneous. Nonetheless, OHCs must prioritize robust team engagement as a fundamental aspect of patient satisfaction, steering clear of inefficiencies like relying solely on one doctor to address patient queries. Concurrently, team leaders should cultivate a positive atmosphere, encourage active member involvement during OTCs, and deliver better service to patients.

### 5.4. Limitations and Future Directions

This study has several limitations. First, this study only scrutinizes the mediators between OMT engagement and patient satisfaction from the perspective of semantic features. Future research can explore more factors that can illustrate the mechanisms between OMT engagement and patient satisfaction. Second, this study examines how OMT engagement impacts patient satisfaction through two kinds of semantic features (language concreteness and emotional intensity). Future research can attempt to explore more semantic features embedded in OMT-patient communication that can mediate the relationship between OMT engagement and patient satisfaction. Third, this study only considers patient satisfaction as a patient outcome. More outcomes such as patient well-being and repurchase behavior of OMT service can be investigated in future research to enrich the literature.

## 6. Conclusions

Online medical teams (OMTs) have enabled a new mode of online healthcare service. This study explores the underlying mechanism of how OMTs’ engagement influences patient satisfaction through the lens of semantic features. We also scrutinize the moderating effects of multiple specializations on the link between OMTs’ engagement and semantic features. This study is one of the few to investigate the impact of OMT engagement on patient satisfaction while exploring the underlying mechanisms from the perspective of semantic features (language concreteness and emotional intensity). By shedding light on the semantic dynamics of online team consultations, our study underscores how OMT engagement fosters linguistic concreteness, facilitating patient comprehension and bolstering satisfaction. Nevertheless, it reveals a nuanced trend wherein heightened OMT engagement correlates with diminished emotional support—a phenomenon possibly attributed to the inherent focus of online team consultations on resolving complex health issues. Additionally, the engagement of doctors from multiple departments amplifies the impact of OMT engagement on both language concreteness and emotional intensity. This study enriches the literature on OMTs and patient satisfaction, offering vital insights for online health platforms aiming to enhance patient satisfaction with OMT services.

## Figures and Tables

**Table 1:** Descriptive statistics.

Variable	N	Mean	Std. Dev.	Min	Median	Max
Patient satisfaction	92,946	0.971	1.907	0.000	0.000	54.90
OMT engagement	92,946	0.349	0.201	0.043	0.333	1.000
Language concreteness	92,946	1.445	0.449	0.114	1.388	4.969
Emotional intensity	92,946	0.087	0.054	0.000	0.081	0.615
Multiple specializations	92,946	0.104	0.305	0.000	0.000	1.000
Sex	92,946	0.448	0.497	0.000	0.000	1.000
Age	92,946	35.44	20.86	0.500	34.00	100.0

**Table 2:** Results of stage 1.

	(1)	(2)	(3)	(4)	(5)	(6)
Variables	Language Concreteness	Language Concreteness	Language Concreteness	Emotional Intensity	Emotional Intensity	Emotional Intensity
OMT engagement		0.025 *	0.022 *		−0.005 ***	−0.004 **
		(0.013)	(0.013)		(0.002)	(0.002)
Multiple specializations		−0.005	−0.448 ***		−0.005 ***	−0.082 ***
		(0.006)	(0.008)		(0.001)	(0.001)
Multiple specializations * Language concreteness			1.412 ***			
			(0.016)			
Multiple specialization * Emotional intensity						0.915 ***
						(0.012)
Sex	−0.005	−0.004	−0.004	−0.000	−0.000	0.000
	(0.003)	(0.003)	(0.003)	(0.000)	(0.000386)	(0.000)
Age	0.001 ***	0.001 ***	0.001 ***	−7.46 × 10^−5^ ***	−7.60 × 10^−5^ ***	−7.27 × 10^−5^ ***
	(0.000)	(0.000)	(9.77 × 10^−5^)	(1.28 × 10^−5^)	(1.28 × 10^−5^)	(1.24 × 10^−5^)
Constant	1.427 ***	1.419 ***	1.417 ***	0.090 ***	0.092 ***	0.092 ***
	(0.004)	(0.006)	(0.006)	(0.001)	(0.001)	(0.001)
Team fixed effects	YES	YES	YES	YES	YES	YES
No. of teams	2122	2122	2122	2122	2122	2122
Observations	92,946	92,946	92,946	92,946	92,946	92,946
R-squared	0.199	0.199	0.262	0.122	0.123	0.179

Notes. Standard errors in parentheses; *** *p* < 0.01, ** *p* < 0.05, * *p* < 0.1.

**Table 3:** Results of stage 2.

	(1)	(2)
Variables	Patient Satisfaction	Patient Satisfaction
Language concreteness		0.097 ***
		(0.014)
Emotional intensity		2.624 ***
		(0.111)
Sex	0.010	0.012
	(0.013)	(0.013)
Age	−0.000	−5.28 × 10^−5^
	(0.000)	(0.000)
Constant	0.973 ***	0.598 ***
	(0.017)	(0.027)
Team fixed effects	YES	YES
No. of teams	2122	2122
Observations	92,946	92,946
R-squared	0.225	0.230

Notes. Standard errors in parentheses; *** *p* < 0.01, ** *p* < 0.05, * *p* < 0.1.

**Table 4:** Mediation check.

Paths	Direct Effect	95%CI	Indirect Effect	95%CI
OMT engagement—Language concreteness—Patient satisfaction	1.360	(1.271, 1.453)	0.013	(0.009, 0.017)
OMT engagement—Emotional intensity—Patient satisfaction	1.394	(1.304, 1.489)	−0.023	(−0.028, −0.017)

**Table 5:** Results of stage 1 (OLS).

	(1)	(2)	(3)	(4)	(5)	(6)
Variables	Language Concreteness	Language Concreteness	Language Concreteness	Emotional Intensity	Emotional Intensity	Emotional Intensity
OMT engagement		0.049 ***	0.028 ***		−0.012 ***	−0.011 ***
		(0.008)	(0.007)		(0.001)	(0.001)
Multiple specializations		−0.010 *	−0.537 ***		−0.001 **	−0.085 ***
		(0.005)	(0.007)		(0.001)	(0.001)
Multiple specializations * Language concreteness			1.617 ***			
			(0.016)			
Multiple specializations * Emotional intensity						0.999 ***
						(0.012)
Sex	0.010 ***	0.010 ***	0.005 *	−0.001 **	−0.001 **	−0.000
	(0.003)	(0.003)	(0.003)	(0.000)	(0.000)	(0.000)
Age	0.001 ***	0.001 ***	0.001 ***	0.000 ***	0.000 ***	9.85 × 10^−5^ ***
	(7.06 × 10^−5^)	(7.06 × 10^−5^)	(6.71× 10^−5^)	(8.52 × 10^−6^)	(8.51× 10^−6^)	(8.19 × 10^−6^)
Constant	1.393 ***	1.376 ***	1.390 ***	0.084 ***	0.088 ***	0.088 ***
	(0.003)	(0.004)	(0.004)	(0.000)	(0.001)	(0.000)
Observations	92,946	92,946	92,946	92,946	92,946	92,946
R-squared	0.004	0.005	0.102	0.002	0.004	0.078

Notes. Standard errors in parentheses; *** *p* < 0.01, ** *p* < 0.05, * *p* < 0.1.

**Table 6:** Results of stage 2 (OLS).

	(1)	(2)
Variables	Patient Satisfaction	Patient Satisfaction
Language concreteness		0.125 ***
		(0.014)
Emotional intensity		2.275 ***
		(0.117)
Sex	0.067 ***	0.068 ***
	(0.013)	(0.013)
Age	−0.002 ***	−0.002 ***
	(0.000)	(0.000)
Constant	1.005 ***	0.640 ***
	(0.014)	(0.025)
Observations	92,946	92,946
R-squared	0.001	0.006

Notes. Standard errors in parentheses; *** *p* < 0.01, ** *p* < 0.05, * *p* < 0.1.

**Table 7:** Results of stage 1 (new controls).

	(1)	(2)	(3)	(4)	(5)	(6)
Variables	Language Concreteness	Language Concreteness	Language Concreteness	Emotional Intensity	Emotional Intensity	Emotional Intensity
OMT engagement		0.061 ***	0.028 ***		−0.006 ***	−0.006 ***
		(0.011)	(0.011)		(0.001)	(0.001)
Multiple specializations		−0.012 **	−0.535 ***		−0.003 ***	−0.087 ***
		(0.006)	(0.007)		(0.001)	(0.001)
Multiple specializations *			1.611 ***			
Language concreteness			(0.016)			
Multiple specializations *						0.994 ***
Emotional intensity						(0.012)
Sex	0.002	0.002	−0.001	−0.001 ***	−0.001 ***	−0.001 **
	(0.003)	(0.003)	(0.003)	(0.000)	(0.000)	(0.000)
Age	0.001 ***	0.001 ***	0.001 ***	0.000 ***	9.88 × 10^−5^ ***	9.62 × 10^−5^ ***
	(7.04 × 10^−5^)	(7.04 × 10^−5^)	(6.69 × 10^−5^)	(8.52 × 10^−6^)	(8.52 × 10^−6^)	(8.20 × 10^−6^)
Price	−0.000 ***	−0.000 ***	−0.000 ***	−6.90 × 10^−5^ ***	−5.92 × 10^−6^ ***	−4.02 × 10^−6^ **
	(1.70 × 10^−5^)	(1.71 × 10^−5^)	(1.62 × 10^−5^)	(2.06 × 10^−6^)	(2.07 × 10^−6^)	(1.99 × 10^−6^)
Team size	0.002 ***	0.005 ***	0.004 ***	0.001 ***	0.001 ***	0.001 ***
	(0.001)	(0.001)	(0.001)	(7.29 × 10^−5^)	(0.000)	(9.89 × 10^−5^)
Leader response	0.038 ***	0.035 ***	0.041 ***	0.000	0.001 **	0.001 **
	(0.003)	(0.003)	(0.003)	(0.000)	(0.000)	(0.000)
Tenure	−0.022 ***	−0.022 ***	−0.022 ***	0.002 ***	0.002 ***	0.002 ***
	(0.001)	(0.001)	(0.001)	(0.000)	(0.000)	(0.000)
Constant	1.475 ***	1.443 ***	1.455 ***	0.072 ***	0.075 ***	0.076 ***
	(0.007)	(0.009)	(0.009)	(0.001)	(0.001)	(0.001)
Observations	92,946	92,946	92,946	92,946	92,946	92,946
R-squared	0.013	0.014	0.110	0.006	0.007	0.080

Notes. Standard errors in parentheses; *** *p* < 0.01, ** *p* < 0.05, * *p* < 0.1.

**Table 8:** Results of stage 2 (new controls).

	(1)	(2)
Variables	Patient Satisfaction	Patient Satisfaction
Language concreteness		0.124 ***
		(0.014)
Emotional intensity		2.400 ***
		(0.116)
Sex	0.094 ***	0.097 ***
	(0.013)	(0.013)
Age	−0.002 ***	−0.003 ***
	(0.000)	(0.000)
Price	0.002 ***	0.002 ***
	(7.19 × 10^−5^)	(7.18 × 10^−5^)
Team size	−0.044 ***	−0.047 ***
	(0.003)	(0.003)
Leader response	0.383 ***	0.377 ***
	(0.015)	(0.014)
Tenure	0.008	0.006
	(0.005)	(0.005)
Constant	0.751 ***	0.396 ***
	(0.031)	(0.038)
Observations	92,946	92,946
R-squared	0.017	0.023

Notes. Standard errors in parentheses; *** *p* < 0.01, ** *p* < 0.05, * *p* < 0.1.

## Data Availability

The data that support the findings of this study are available from the corresponding author upon reasonable request.
